# Microarc Oxidation Coating Combined with Surface Pore-Sealing Treatment Enhances Corrosion Fatigue Performance of 7075-T7351 Al Alloy in Different Media

**DOI:** 10.3390/ma10060609

**Published:** 2017-06-02

**Authors:** Hui-Hui Yang, Xi-Shu Wang, Ya-Ming Wang, Yan-Ling Wang, Zhi-Hao Zhang

**Affiliations:** 1 Department of Engineering Mechanics, School of Aerospace Engineering, AML, Tsinghua University, Beijing 100084, China; yanghh14@mails.tsinghua.edu.cn (H.-H.Y.); vipsophia@126.com (Y.-L.W.); zzh6230@163.com (Z.-H.Z.); 2 Institute for Advanced Ceramics, Harbin Institute of Technology, Harbin 150001, China; wangyaming@hit.edu.cn

**Keywords:** Al alloy, corrosion fatigue, microarc oxidation coating, pore-sealing treatment, microscopic analysis

## Abstract

Rotating bending fatigue tests have been performed to evaluate the corrosion fatigue performance and its influence factors of 7075-T7351 Al alloy in different media, namely air and a 5.0 wt % NaCl aqueous solution. All samples were coated by microarc oxidation (MAO) coating technology; some samples were followed by an epoxy resin pore-sealing treatment. Microscopic analyses of the surfaces and fracture cross-sections of samples were carried out. The results reveal that the sample with a MAO coating of 10 μm thickness and pore-sealing treatment by epoxy resin possesses optimal corrosion fatigue performance in the different media. The MAO coating with a pore-sealing treatment significantly improves the corrosion fatigue limit of 7075-T7351 Al alloy.

## 1. Introduction

There has been growing interest in light metals such as aluminium, magnesium and titanium alloys, which have been widely applied to aeronautic and automotive industries due to their high strength-to-weight ratios [1]. However, poor abrasion resistance and inferior corrosion resistance have limited their extensive applications [1].

Microarc oxidation (MAO) coating technology is a relatively new surface engineering technique that has significantly advanced in the last two decades [2]. MAO coating technology has proved to be suitable for enhancing abrasion resistance and corrosion resistance of light metals (Al, Mg and Ti alloys) by applying spark or arc plasma micro-discharges to an aqueous solution; this ionizes gaseous media from the solution so that oxides are easily formed on the surface of the light metals through the plasma chemical interactions [1,2,3,4]. In most cases, the adhesive strength between the MAO coating and the substrate is higher than for other methods (such as magnetron sputtering) [5,6]. Therefore, the interfacial failure behavior is very difficult to generate. Nevertheless, for a typical structural material subjected to the MAO process, when compared to uncoated counterparts, the mechanical performance of a MAO coated metal may be different. For instance, the fatigue performance of MAO coated metals is significantly reduced [7,8,9,10,11], according to reported results in the last decade. On the other hand, fatigue behavior is not modified by the MAO process in both Ti-6Al-7Nb and CP-Ti, when compared to the samples without surface modification [12]. Therefore, it is important to consider the effect of the MAO coating on the fatigue performance of MAO coated metals and alloys in their possible engineering applications, such as biomedicine [13], textile machine building, gas-oil industry, engine industry, electrical engineering and electronics etc. [7]. Yerokhin et al. [7,8] studied the effect of coating thicknesses (7 and 15 μm) on fatigue properties of MAO treated Mg alloy (2% Al, 1% Zn, 0.2% Mn and Mg balance). Their results demonstrated that MAO coatings reduced the bending fatigue limit of Mg alloy by no more than 10%. Rajasekaran et al. [9] investigated the effect of different MAO coating thicknesses (0, 40 and 100 μm) on plain fatigue and fretting fatigue behavior of AA6063 alloy. Their results indicated that the cyclic number under the applied stress amplitude (160 MPa) is about 2.0 × 10^6^ cycles for uncoated samples in air, but for samples with coating thicknesses of 40 and 100 μm, the cyclic numbers under the same stress amplitude in air are 2.0 × 10^5^ and 1.1 × 10^5^, respectively. The fatigue performance of AA6063 alloy with a MAO coating has evidently been reduced. Lonyuk et al. [11] also found that the fatigue limit of 7575-T6 Al alloy with coating thicknesses of 14, 35 and 65 μm was reduced by 30%, 51% and 58% respectively, when compared with uncoated samples. 

In addition to the MAO coating thickness, the residual stress and preparation methods of oxidation coating also play important roles in the fatigue performance of light metals [11]. However, due to the structural complexity and various influence factors of a coating-substrate structure, the more detailed failure and corrosion fatigue damage mechanisms are still unclear, and are especially lacking countermeasures for improving the corrosion fatigue resistance. In addition, nearly no experimental data was available regarding the influences of MAO coatings on corrosion fatigue behaviors of light metals [1,2,3,4,11]. Wang et al. [14,15] reported that MAO coating with a pore-sealing treatment can improve the mechanical fatigue performance of 2024-T4 Al alloy in air; this is because the inescapable thermal-cracks and shrinkage cavities on MAO coating layers of Al alloys are filled using the pore-sealing treatment by epoxy resin. However, the effect of MAO coating with a pore-sealing treatment on the corrosion fatigue performance of Al alloy has not been studied.

To validate that the pore-sealing treatment can also improve the corrosion fatigue performance of Al alloy with MAO coating, effects of the different MAO coating thicknesses (0, 10, 15, 20 and 30 μm), residual stress, microstructure and corrosion media on the fatigue performance of typical 7075-T7351 Al alloy samples in both air and a 5.0 wt % NaCl aqueous solution were experimentally investigated in this work. 

## 2. Materials and Experimental Methods

### 2.1. Coating Preparation

The MAO pretreated samples were carefully prepared, as for previous studies [1,3,7,16,17], in accordance with the processing conditions listed in Table 1. The electrolyte based on alkaline silicate was prepared from the solution of Na_2_SiO_3_, NaOH and (NaPO_3_)_6_ in distilled water. The current density is the principal factor to be controlled in MAO processing. Normally, to reach the required conditions for plasma electrolysis, the current density is set between 0.01 and 0.30 A/cm^2^. A 150 kW MAO device provides the voltage waveforms, and the pulse parameters (pulse duration, voltage amplitude and duty cycle) can be adjusted independently [7,16,17,18]. Electrical parameters were set as follows: voltage at 600 V, frequency at 600 Hz and duty cycle at 10.0%. The temperature of the electrolyte was maintained below 50 °C and controlled by a cooling system. The different coating thicknesses of samples (10, 15, 20 and 30 μm) were fabricated by MAO treatment under different anodizing oxidation times, as shown in Table 1. Subsequently, the impregnation of epoxy resin was carried out by manual spraying in an ambient temperature followed by drying in the oven at 180 °C. Therefore, the micro-pores or micro-cracks on the coating surface were sealed by epoxy resin (Phoenix WSR6101) [14,15,19]. The as-coated samples with a pore-sealing treatment were washed with distilled water and dried in air.

### 2.2. Experimental Processes

7075-T7351 Al alloy, with a nominal chemical composition (wt %) of 5.89Zn, 2.48Mg, 1.59Cu, 0.22Cr, 0.06Fe, 0.02Ti and a balance of Al, was used as the substrate in the rotating bending fatigue test. The samples were coated using the MAO technology, both with and without a pore-sealing treatment by epoxy resin; a benchmark test was performed on the sample without an MAO coating. The mechanical properties of the substrate (7075-T7351 Al alloy) and MAO coating are listed in Table 2. 

**Figure 1:**
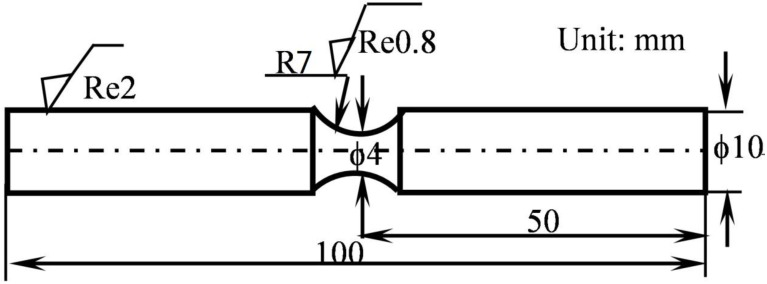
Shape and size sketch of specimen (unit: mm). The picture is not clear, please provide a high-resolution figure.

**Figure 2:**
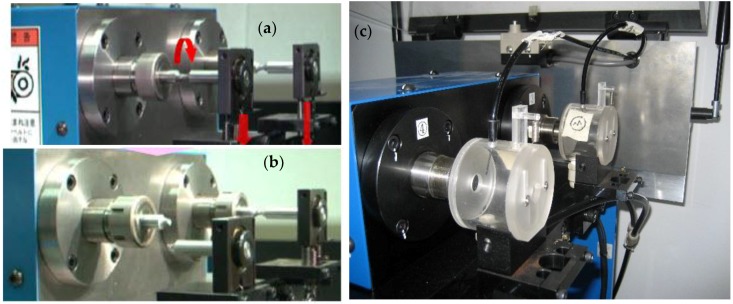
Fatigue testing loading modes for air or corrosive media: (**a**) and (**b**) mechanical loading in air, and (**c**) mechanical loading in corrosive media.

Prior to the MAO coating process, samples were cut from a large plate of 7075-T7351 Al alloy into particular sizes and geometries, as depicted in Figure 1. Each sample had a predefined circular notch in the center to achieve a stress concentration factor of approximately 1.08; we intentionally controlled the corrosive liquid to drop into the circular notch, as shown in Figure 2. All the surfaces of samples were shaped to meet the required dimensions by turning and grinding; they were then smoothly grinded using SiC abrasive paper (2000 grit) to achieve a surface roughness *Re* of about 0.6–0.8 μm. After the mechanical grinding, specimens were cleaned using acetone in an ultrasonic cleaner, and dried for a minimum of 25 min. Subsequently, all the samples were covered with MAO coatings of different thicknesses, with some samples being followed by a pore-sealing treatment using epoxy resin. 

The samples were loaded in the cantilever under the typical loading *W*, as shown in Figure 2. The applied stress amplitude (σ, MPa) in the rotating bending fatigue tests was estimated as follows:

(1) $$\sigma =\;\frac{32g\alpha LW}{\pi d^{3}}$$

In Equation (1), *d* is the diameter of the gauge section (i.e., 4 mm, as shown in Figure 1), *g* is the acceleration of gravity (9.8 m/s^2^), K is the stress concentration factor (1.08), *L* is the distance from the gauge section to the end at which the load is applied (about 40.5 mm for a standard sample) and *W* is the applied load (kg). All rotating bending fatigue tests were controlled at a stress ratio of *R* = −1 and at a rotating frequency of approximately 55 Hz [14,20,21]. Corrosion media, including air and a 5.0 wt % NaCl aqueous solution, were used in these tests. In the corrosion fatigue test, the corrosive liquid was dripped at a rate of 1.6 mL/min.

### 2.3. Residual Stress Measurement

The residual stresses in the MAO coatings of different thicknesses were measured using $sin^{2}\Psi$ (X-ray) diffraction approach for a diffracted plane (311) at *θ* = 67.00°. The measurements were carried out at four different *Ψ* angles (0.00, 24.20, 35.30 and 45.0°), with a scan step of 0.10° and exposure time of 0.50 s. The values of residual compressive stress were calculated as follows [2,11,22]:

(2) $$\sigma_{r}=\frac{E}{2\left(1+\mu \right)}\cot\theta_{0}\frac{\pi }{180}\frac{\partial \left(2\theta_{\Psi }\right)}{\partial \left(sin^{2}\Psi \right)}$$

In Equation (2), *E* is Young’s modulus of coating (*γ*-Al_2_O_3_) and μ is Poisson’s ratio, given as 253.00 GPa and 0.24, respectively [11]. *θ_0_* = 72.50° is the scanning angle, while *Ψ* = 0. Table 3 shows the residual compressive stress of MAO coatings of different thicknesses with a pore-sealing treatment, according to Equation (2). The results indicate that the residual compressive stress increases with the increasing of MAO coating thickness. 

## 3. Results

### 3.1. Surface Morphology of MAO Coated Samples

**Figure 3:**
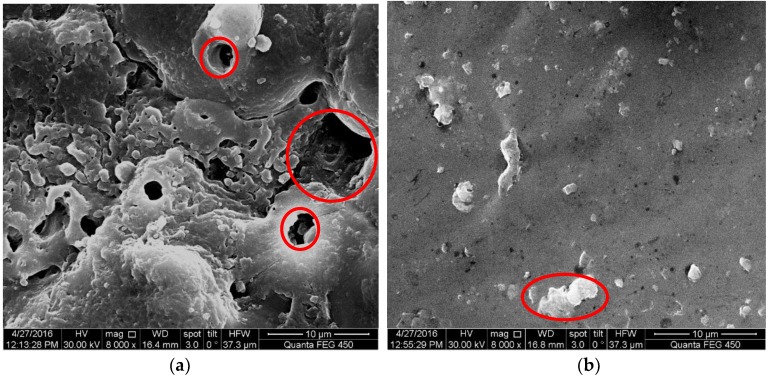
SEM micrograph of surface structures of MAO coated samples (scale bar 10 μm): (**a**) without the pore-sealing treatment, and (**b**) with the pore-sealing treatment.

Figure 3 shows surface structures of MAO coated samples, respectively without and with a pore-sealing treatment. The shrinkage cavities and thermal-cracks on the surface of samples without a pore-sealing treatment are clearly shown in Figure 3a; the maximum diameter of cavities was about 10 μm and the average diameter was about 2–3 μm. For the surface of samples with a pore-sealing treatment, these shrinkage cavities were filled by the epoxy resin, however a few agglomerates of epoxy resin were found as shown in Figure 3b. The surface of the MAO coating with a pore-sealing treatment was clearly much smoother than for that without. Therefore, for the surface with a pore-sealing treatment, the stress concentration was also reduced. Furthermore, the surface fatigue crack initiation resistance or the early stage of fatigue crack propagation resistance could be further enhanced.

**Figure 4:**
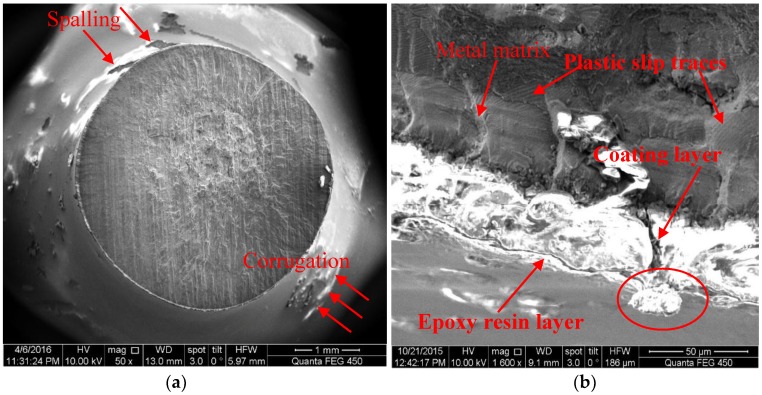
SEM micrograph of corrosion fatigue cracking characteristics of MAO coated samples with pore-sealing treatment (*h* = 20 μm, σ = 102 MPa, *N_f_* = 201,100) in a 5.0 wt % NaCl solution: (**a**) scale bar 1 mm, and (**b**) scale bar 0.05 mm.

Figure 4 shows the typical corrosion fatigue fracture characteristics of a MAO coated sample with pore-sealing treatment in a 5.0 wt % NaCl solution. These characteristics include: (1) The corrugation and spalling behaviors were clearly seen on the surface of the sample, as depicted in Figure 4a (marked by arrows), while they were not found on the surface of samples without a pore-sealing treatment [14]. (2) The corrosion fatigue crack initiation occurred preferentially at the position of maximum accumulation of epoxy resin, close to the primordial shrinkage cavities (marked by the elliptical ring), in which one crack penetrated from the coating to the substrate—as shown in Figure 4b. However, the interfacial crack between the coating and the substrate was not found in Figure 4b, which further validated that using the MAO coating technology, the adhesion strength was high [5,6]. (3) A large number of plastic slip striations appeared nearby the sub-surface of the substrate in Figure 4b. The directions of plastic slip striations were 30–50° tilted to the rotation direction of the sample, which hinted that in the fracture surface, there was not only a normal stress but also a shear stress; this made it easy to cause the crack initiation and propagation [15]. The striations and directions, as shown in Figure 4b, indicated a discontinuous crack propagation mechanism: an electrochemical attack on plastic deformation zones [23]. Obviously, the reduction in the accumulation of epoxy resin during the pore-sealing process can turn down the occurrence probability of penetrated cracks so that the corrosion fatigue performance of the coating-substrate could be improved. References should be cited in numerical order.

### 3.2. Corrosion Fatigue Performance of Coating-Substrate in the Different Conditions

**Figure 5:**
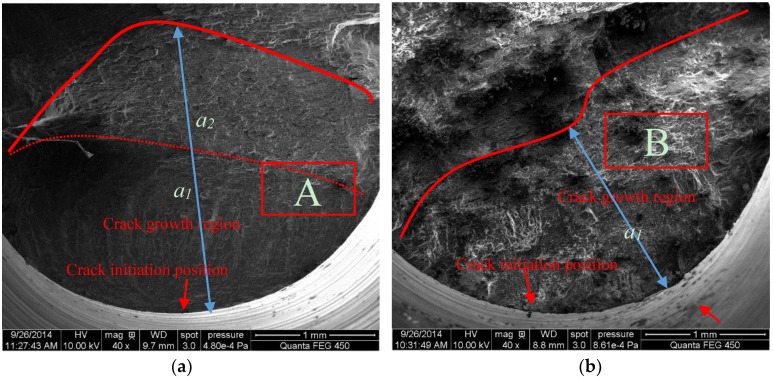

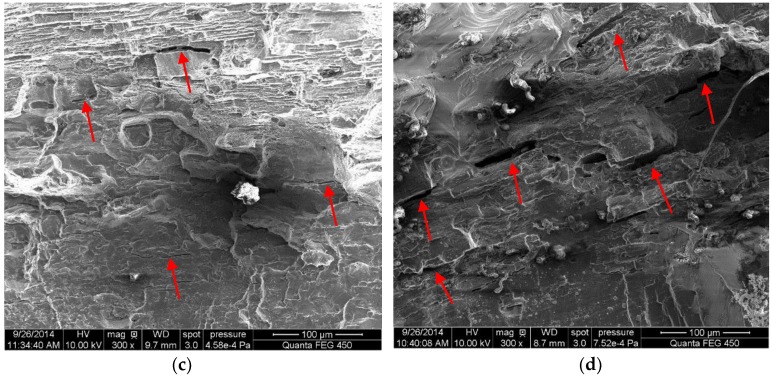
The typical SEM micrograph of fatigue fracture characteristics (*h* = 10 μm) without the pore-sealing treatment (253 MPa) in different environmental media: (**a**) in air, (**b**) in a 5.0 wt % NaCl solution, (**c**) magnified view of area A, and (**d**) magnified view of area B.

Figure 5 shows the typical corrosion fracture characteristics of 7075-T7351 Al alloy with a MAO coating without the pore-sealing treatment. There are clear differences between fracture surfaces in air and in a 5.0 wt % NaCl aqueous solution, as shown by Figure 5a,b. In air, the fatigue crack propagation region can be divided into two (regions *a_1_* and *a_2_*), and the fatigue crack initiation position can be identified at the red arrow mark (Figure 5a). In NaCl solution (Figure 5b) the fatigue crack propagation length *a_1_* is smaller than *a_1_* + *a_2_* (Figure 5a); however, this does not indicate that the corrosion fatigue crack growth rate in NaCl solution is less than in air because of the electrochemistry reaction, and coupled action of corrosive liquid and stress in NaCl solution [15]. In addition, the corrosion fatigue crack initiation can be identified by the red arrow marks (Figure 5b). On the other hand, the typical magnifying images in air and a 5.0 wt % NaCl solution show the opening displacement of second cracks and concave-convex characteristics in the fatigue crack propagation processes (Figure 5c,d), in which the opening displacement of second cracks in air is less than in a 5.0 wt % NaCl solution, and concave-convex patterns are different between the two media. The microscopic fracture characteristics are also in good agreement with previous works [24,25]. A similar trend was observed for thicker coatings.

**Figure 6:**
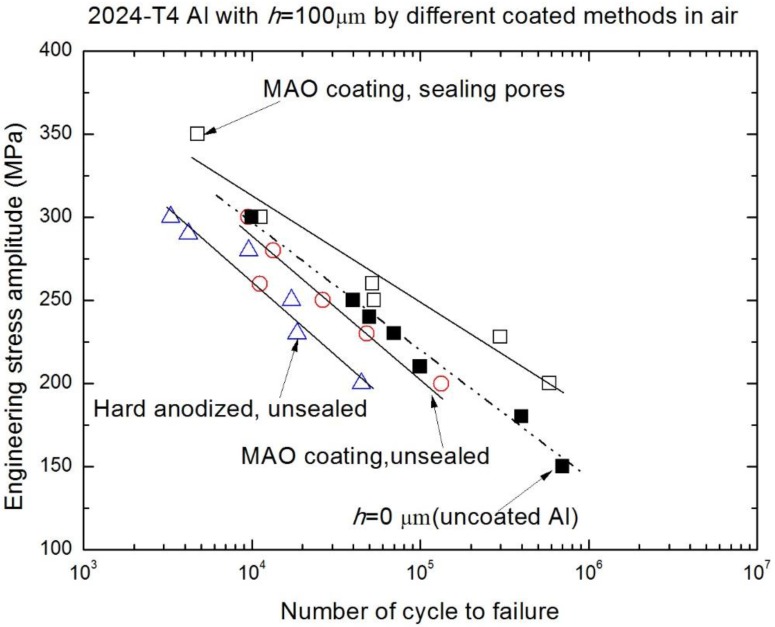
S-N curves of 2024-T4 Al alloy using the different coating preparation methods [13].

**Figure 7:**
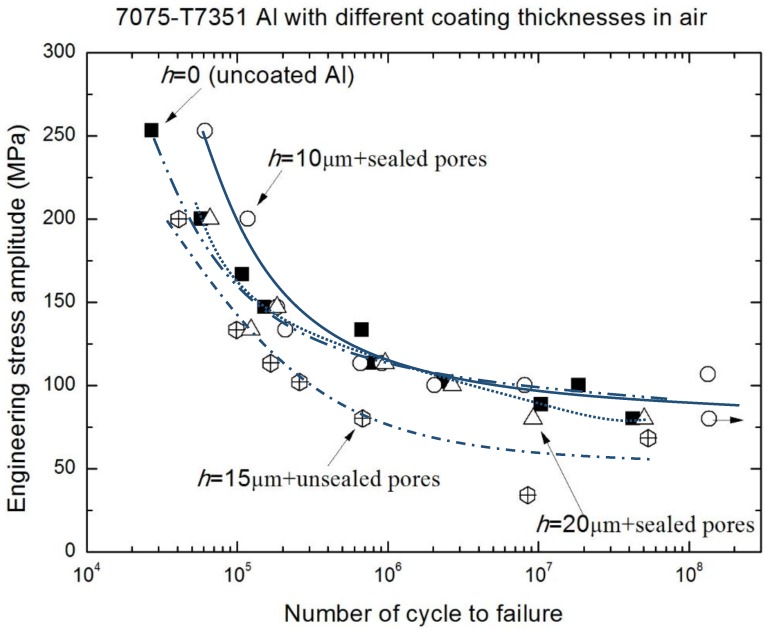
S-N curves of 7075-T7351 Al alloy with different MAO coating thicknesses in air.

Figure 6 and Figure 7, in which the fatigue data for 2024-T4 Al alloy was taken from previous work [14], reflect that the fatigue performances of both the high strength Al alloy (7075-T7351) and low strength Al alloy (2024-T4) in air have been improved by the MAO coatings with a pore-sealing treatment. In addition, the effect of the coating preparation method on the fatigue performance of 2024-T4 Al alloy is revealed in Figure 6. Figure 7 shows the applied stress amplitude σ versus cycles to failure (S-N curves) of 7075-T7351 Al alloy with different MAO coating thicknesses in air; compared with both the alloy of *h* = 15 μm coating under no pore-sealing treatment and the uncoated alloy, the *h* = 10 μm MAO coated alloy under the pore-sealing treatment improved within 10^6^ cycles. This indicates that the pore-sealing treatment can effectively improve the fatigue performances of both the high strength Al alloy (7075-T7351) and low strength Al alloy (2024-T4). In addition, the change trends of S-N curves in Figure 6 and Figure 7 agree well with previous studies [4,7,8,9,10,11,14,15,26,27]. For example, it has been reported that a hard-anodized ceramic coating led to about a 75% reduction of the fatigue strength for 7475-T6 Al alloy, and a MAO coating without the pore-sealing resulted in about a 58% reduction of the fatigue strength for 7475-T6 Al alloy [11]. It can generally be accepted that these reductions are due to thermal micro-cracks and micro-shrinkage cavities on the surface and the brittle property of the coating [11]. Therefore, once thermal micro-cracks and micro-shrinkage cavities are filled by the pore-sealing technology, the mechanical fatigue performance of 7075-T7351 Al alloy can be enhanced.

**Figure 8:**
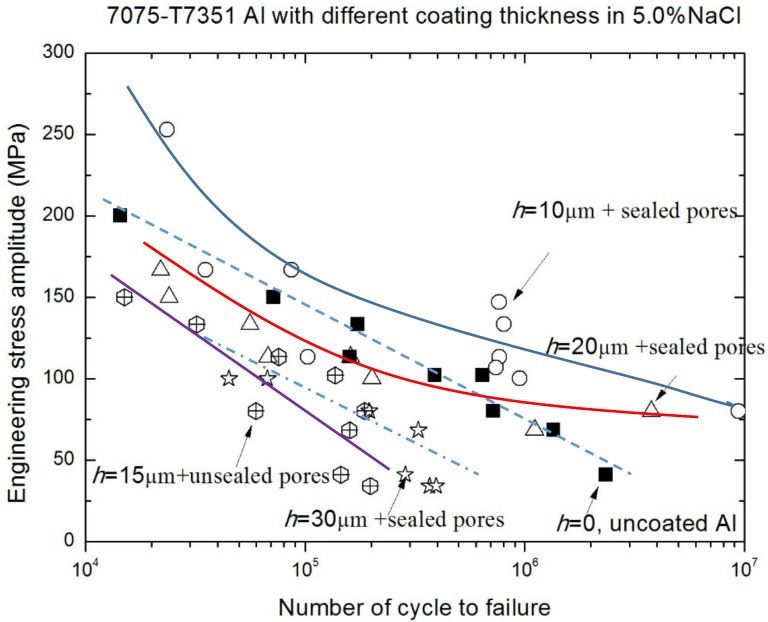
S-N curves of 7075-T7351 Al alloy with different MAO coating thicknesses in a 5.0 wt % NaCl solution.

For 7075-T7351 Al alloy, other significant influence factors of fatigue performance are the MAO coating thickness and the environmental medium. The results in typical environmental medium (a 5.0 wt % NaCl aqueous solution) are shown in Figure 8. Although there are some scattered data in the corrosion fatigue test, the overall trend of S-N curves for 7075-T7351 Al alloy with different MAO coating thicknesses (marked by different lines) can be clearly seen. With the increasing of MAO coating thickness, the corrosion fatigue performance reduces. In NaCl solution, the difference of corrosion fatigue performance between *h* = 10 μm and *h* = 20 μm is more pronounced, compared with that in air (Figure 7). This means that the corrosion fatigue performance of 7075-T7351 Al alloy is sensitive to the MAO coating thickness. When compared to the other alloys (without MAO coating, with a coating of *h* = 15 μm without the pore-sealing treatment, and with a coating of *h* = 20 μm and *h* = 30 μm with the pore-sealing treatment), the corrosion fatigue performance of the *h* = 10 μm MAO coated alloy with the pore-sealing treatment is still optimal. In addition, Table 3 shows that with the increasing of MAO coating thickness, the residual compressive stress in the coating also increases. Therefore, the effect of residual compressive stress on corrosion fatigue performance of 7075-T7351 Al alloy with a MAO coating can be equivalent to the effect of MAO coating thickness. This reflects that the corrosion fatigue performance of 7075-T7351 Al alloy with a MAO coating can also be enhanced using the pore-sealing treatment, but that there is an optimal MAO coating thickness that is interrelated to the residual compressive stress level in the coating. For coatings thinner than 10 µm, there are few or no cracks after the pore-sealing treatment, which leads to a better fatigue performance. For coatings thicker than 10 µm, cracks in the inner coating are not filled well, leading to a lower fatigue resistance.

**Figure 9:**
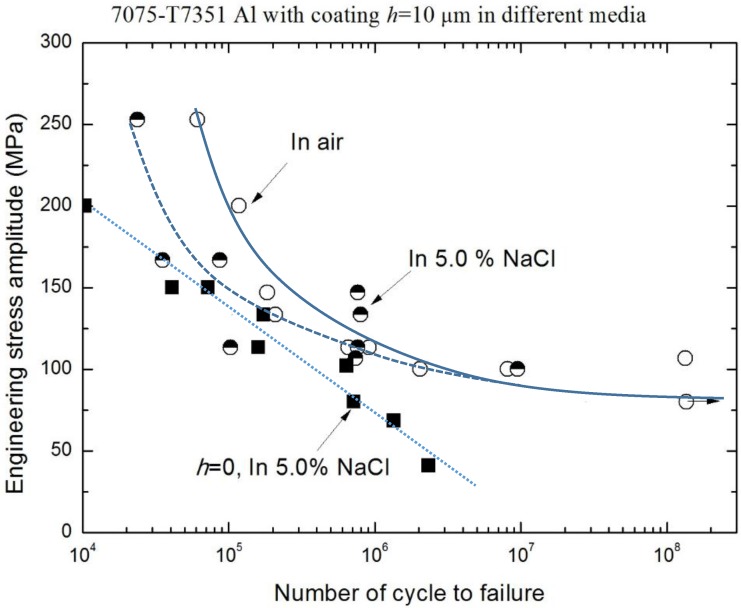
S-N curves of 7075-T7351 Al alloy with the same thickness (*h* = 10 μm) in different media.

**Figure 10:**
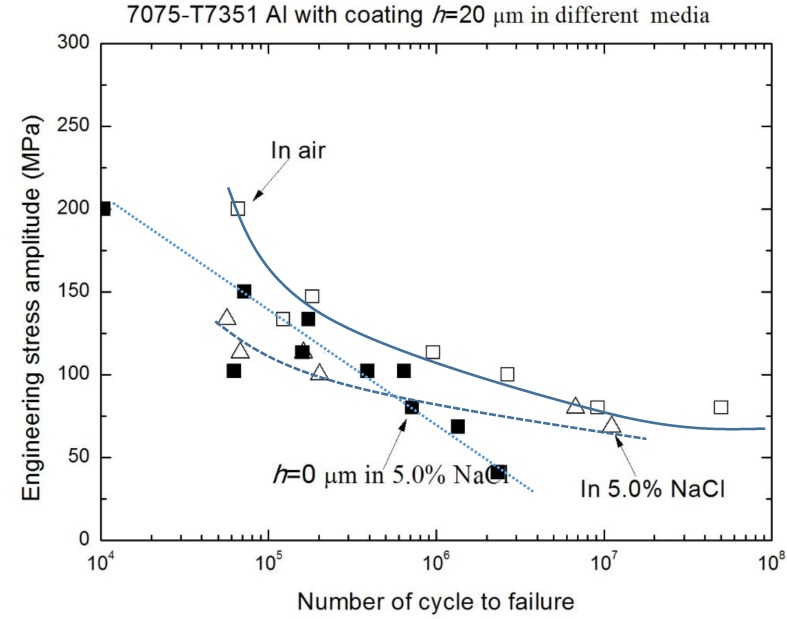
S-N curves of 7075-T7351 Al alloy with the same thickness (*h* = 20 μm) in different media.

Figure 9 and Figure 10 show the effect of an environmental medium on the fatigue performance of 7075-T7351 Al alloy with *h* = 10 μm and *h* = 20 μm, respectively. As for *h* = 10 μm, the fatigue limits (σ_−1_) in air and in a 5.0 wt % NaCl aqueous solution are approximately the same (about 102 MPa), as shown in Figure 9. This hints that in the low stress level, a MAO coating with the pore-sealing treatment makes it difficult to produce the corrugation or spalling behavior; the protective layer plays an important role in inhibiting corrosion fatigue crack initiation. However, in the high stress level (over 150 MPa), the effect of an environmental medium on the fatigue performance of 7075-T7351 Al alloy cannot be ignored, as shown in Figure 9. That is, in the high stress level, the corrugation or spalling behavior and the coupling action between the environmental medium and stress occur easily in the MAO coating layer. Figure 10 shows that for 7075-T7351 Al alloy with *h* = 20 μm, the corrosion fatigue limit in a 5.0 wt % NaCl aqueous solution is slightly smaller than for in air. Likewise, the fatigue performance can be affected by the environmental medium in the high stress level. 

**Figure 11:**
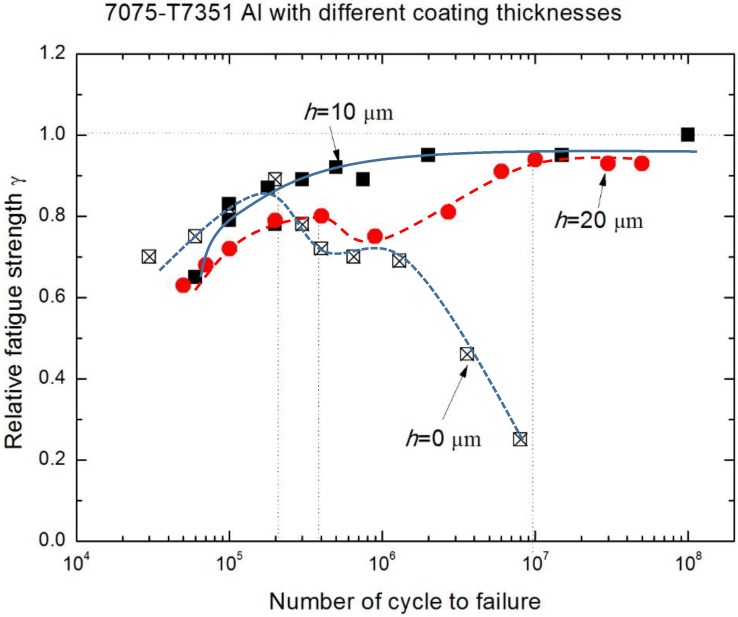
Relative fatigue strength ratio versus cycles to failure of 7075-T7351 Al alloy with different MAO coating thicknesses.

To quantitatively elucidate the effective scope of the environmental medium and MAO coating thickness on the fatigue performance of 7075-T7351 Al alloy, the relative fatigue strength ratio γ (γ = σ_NaCl_/σ_air_) versus cycles to failure was plotted for alloys of different MAO coating thicknesses (h = 10 μm and h = 20 μm) as well as the uncoated 7075-T7351 Al alloy, as shown in Figure 11. A smaller *γ* indicates a higher corrosion fatigue sensitivity. In contrast, the corrosion fatigue resistance is good when *γ* approaches one [25]. Figure 11 indicates that among *h* = 10 μm, *h* = 20 μm and uncoated 7075-T7351 Al alloys, there are different corrosion fatigue behaviors within the range from *N_f_* = 1 × 10^5^ to *N_f_* = 1 × 10^7^. For the uncoated metal, the change trend of *γ* from 2 × 10^5^ to 1 × 10^7^, similar to the result of M.M. Sharma [25], decreases with the increasing of cycles to failure. For the coated metal, the effect of an environmental medium on corrosion fatigue of *h* = 20 μm alloy is more pronounced than for *h* = 10 μm alloy. In addition, the effect on *h* = 20 μm alloy is nonlinear—increasing at the beginning, then decreasing, then finally increasing again. Strong effects of an environmental medium on the fatigue failure of 7075-T7351 Al alloy with *h* = 20 μm occur within the range from 4 × 10^5^ to 2 × 10^6^ cycles; in this range, the coupling action between the environmental medium and the mechanical load should be paid attention to in the future. For the thicker coatings (*h* = 20 μm), cracks in the inner coating are not filled well and electrochemical corrosion becomes severe so that the relative fatigue strength decreases within a certain range. In addition, the MAO coating with the pore-sealing treatment can enhance the corrosion fatigue performance of 7075-T7351 Al alloy, and its corrosion fatigue crack initiation can be attributed to three characteristics: (1) breakdown (corrugation and spalling) of MAO coating with pre-sealing treatment in high stress level, (2) forming pits and causing a stress concentration at the surface or interface, and (3) electrochemical attack on plastic deformation zones where the un-deformed zones act as a large cathode [22,25,28,29]. The evidence from the scanning electron microscopy analysis for these interpretations is given in Section 3.1.

## 4. Conclusions

Fatigue performance of 7075-T7351 Al alloy and its influence factors were investigated through corrosion fatigue tests for uncoated samples and MAO coated samples with and without pore-sealing treatments in environmental media, air and a 5.0 wt % NaCl aqueous solution. The main conclusions are as follows:

1. Through the pore-sealing treatment by epoxy resin, the unfavorable thermal micro-cracks and micro-pores were filled. Therefore, the pore-sealing treatment can also improve the corrosion fatigue performance of 7075-T7351Al alloy with MAO coating.
2. The relative fatigue strength ratio (*γ* = σ_NaCl_/σ_air_) indicates that the major effect of MAO coating thickness on corrosion fatigue behavior occurs within the range from 1 × 10^5^ to 1 × 10^7^. Compared with *γ* of uncoated 7075-T7351 Al alloy, the MAO coating using the pore-sealing treatment can enhance the corrosion fatigue performance.
3. The effect of MAO coating thickness on the corrosion fatigue performance of 7075-T7351 Al alloy suggests an optimizing value, such as *h* = 10 μm. For such a thickness of MAO coating on 7075-T7351 Al alloy, the fatigue limit in a 5.0 wt % NaCl aqueous solution approaches that for in air.
4. For samples of MAO coated alloy with the pore-sealing treatment, microscopic analysis based on SEM images of surfaces and fracture cross-sections provides some evidence of corrosion fatigue cracks forming; these are breakdowns (corrugation and spalling) in high stress level, forming pits that cause a stress concentration and electrochemical attack on plastic deformation zones where the non-deformation areas act as a large cathode.

## Figures and Tables

**Table 1:** Experimental conditions for preparation of microarc oxidation (MAO) coating.

Applied Voltage (V)	Electrolyte Composition	Treatment Time (min)	Coating Thickness, h (μm)	Coating Composition
600	Na_2_SiO_3_, 8 gL^−1^ NaOH, 1 gL^−1^ (NaPO_3_)_6_, 20 gL^−1^	13	10	*γ*-Al_2_O_3_
		19	15	*γ*-Al_2_O_3_
		25	20	*γ*-Al_2_O_3_
		38	30	*γ*-Al_2_O_3_

**Table 2:** Major mechanical properties of substrate and MAO coating.

Material	*E* (GPa)	μ	σ_0.2_ (MPa)	σ_b_ (MPa)	δ (%)
7075-T7351 Al	70.5	0.30	465	528	14.6
*γ*-Al_2_O_3_	253	0.24			

**Table 3:** Residual stress in the coating of samples with MAO coatings of different thicknesses (after a pore-sealing treatment).

Coating Thickness (μm)	Residual Stress (MPa)	Deviation (MPa)
10	−198.27	±11
20	−263.74	±13
30	−446.90	±28
